# Collaborative Bearing Mechanism of Sustainable Coal Gangue Geopolymer Gel Backfill–Rock Combination Under Compression

**DOI:** 10.3390/gels12060517

**Published:** 2026-06-10

**Authors:** Peng Zhang, Zhi Wen, Fei Wang, Cancan Chen

**Affiliations:** School of Water Conservancy and Transportation, Zhengzhou University, Zhengzhou 450001, China; zhangpeng@zzu.edu.cn (P.Z.); 15517100309@163.com (Z.W.); ccchen1365@zzu.edu.cn (C.C.)

**Keywords:** coal gangue, geopolymer gel backfill material, mechanical property, digital image correlation, acoustic emission

## Abstract

Using solid wastes to fabricate sustainable backfill materials for mining engineering is crucial for environmental sustainability worldwide. In this study, the use of coal gangue aggregates as a sustainable alternative to natural aggregates in geopolymer gel backfill materials was explored, which contributes to green mining development. Through uniaxial compression tests, the effects of fine gangue content, mass concentration, and the binder content of geopolymer backfill materials on the compressive behavior of coal gangue geopolymer gel backfill–rock combinations (CGBRC) were systematically evaluated. Digital Image Correlation (DIC) and acoustic emission (AE) techniques were employed to reveal the strain field evolution and damage progression of CGBRC. Results show that as the content of fine coal gangue increases, the compressive strength first increases and then decreases. Compared with the compressive strength at a 20% content, the compressive strength at a 40% content increased by 33.2%, while the elastic modulus increased by 11.2%. Meanwhile, with the increase in mass concentration and binder content, the compressive strength and elastic modulus of coal gangue geopolymer filling materials show an increasing trend, reaching peak values at 86% mass concentration and 32% binder content, respectively. The strain concentration zones mainly form near the backfill interface, with propagation paths governed by backfill strength. Damage evolution undergoes three stages including rapid accumulation during compaction, gradual development in the elastic-plastic stage, and abrupt acceleration at failure. The interfacial debonding behavior is primarily influenced by the strength difference between the backfill and surrounding rock. Specimen failure is dominated by brittle shear fracture, categorized into three modes based on crack paths relative to the backfill, which include penetrating backfill failure, axisymmetric interface failure, and centrally symmetric interface failure. These findings offer theoretical and technical support for coal gangue resource utilization and green mining practices, advancing sustainable solid waste management.

## 1. Introduction

Coal mining activities disrupt the original stress equilibrium of strata, inducing geological hazards such as surface subsidence that severely threaten mining safety and cause ecological damage. Simultaneously, the massive accumulation of coal gangue, a byproduct of coal mining, not only occupies land resources but also leads to environmental pollution in the atmosphere, water bodies, and soil [1]. Utilizing coal gangue in backfill materials allows for solid waste recycling while replacing natural aggregates to reduce costs, which makes it highly significant for mine ecological restoration [2]. Therefore, in-depth research on the engineering applications of backfill materials is essential.

To address the dual challenges of coal gangue accumulation and natural construction material shortages, extensive studies have been conducted on coal gangue resource utilization [3,4]. In cement-based material applications, coal gangue processed through crushing and sorting is used as high-quality aggregates or mineral admixtures. Particle size distribution of coal gangue is significantly improved through optimized crushing and screening processes, which enhances the compactness and compressive strength of backfill material. Experimental results confirm that mechanical requirements for road construction and other engineering applications are fully met by coal gangue aggregates [3,5]. In cement production, coal gangue containing trace elements such as sulfur and vanadium is used to substitute traditional raw materials for clinker production. Solid waste is consumed and carbon emissions during calcination are reduced through this approach, offering notable environmental benefits [4]. Meanwhile, novel fiber-reinforced eco-friendly geopolymer concrete has been developed and successfully applied in infrastructure systems such as concrete pipelines, verifying the engineering application potential and sustainability advantages of geopolymer composites in practical engineering [6]. Beyond these applications, coal gangue has also shown considerable potential as a sustainable aggregate resource for mine backfilling and underground engineering projects. Consequently, increasing attention has been devoted to understanding the interaction mechanisms between filling materials and surrounding rock masses.

In underground engineering projects, cavity defects are commonly repaired using cement-based filling materials. Accordingly, extensive studies have been conducted on the interaction between filling materials and surrounding rock. Previous research has shown that filling materials can effectively suppress tensile stress, increase crack initiation stress, and improve the load-bearing capacity of defective rock masses [7,8]. The mechanical performance of filled composite specimens is strongly affected by the properties of the filling material. In particular, increasing filling material strength and aggregate particle size can significantly enhance the uniaxial compressive strength and elastic modulus of the composite structures [9]. In addition, defect geometry plays an important role in determining crack propagation and failure characteristics, although its influence on elastic modulus is relatively limited [10,11]. Furthermore, advanced monitoring techniques such as Digital Image Correlation (DIC) have revealed that high-strength filling materials can alter crack initiation patterns, mitigate strain concentration around defects, and improve the overall mechanical behavior of filled rock masses [12]. Overall, existing studies have confirmed the effectiveness of filling materials in enhancing the strength, stiffness, and crack resistance of defective rock masses.

Among various filling materials, coal gangue has attracted increasing attention due to its abundant availability, low cost, and environmental benefits. Consequently, considerable efforts have been devoted to investigating the mechanical behavior and failure characteristics of coal gangue-based backfill–rock composite structures. Research on the engineering applications of coal gangue as cement-based backfill materials has primarily focused on the influence mechanisms of filling ratio, aggregate particle size, and backfill strength on crack propagation behavior and failure mechanisms in composite structures. Study results demonstrate that crack propagation paths and failure modes in backfill–rock combination are significantly regulated by filling ratio and aggregate size [9,13]. Comparative tests confirm that the compressive strength of backfilled specimens is higher than that of unfilled specimens, and a critical compressive strength threshold of approximately 15 MPa is identified, beyond which crack development in combination structures is markedly reduced [7,14,15]. Uniaxial compression tests further reveal the mechanical properties of coal gangue backfill composites depend on composition. A higher coarse aggregate proportion increases the elastic modulus but also brittleness, while gradation optimization reduces macroporosity by 40%, boosting compressive strength by 35.6% [1,16,17]. The microstructural characterization results based on the technology of Computed Tomography (CT) and Mercury Intrusion Porosimetry (MIP) indicate that the gradual accumulation of microdamage in heterogeneous materials is the primary factor controlling sample failure [18].

Although considerable progress has been made in understanding the mechanical behavior of coal gangue-based backfill materials, studies employing geopolymer gels as binders in combination with coal gangue remain relatively scarce. In particular, there is a lack of systematic investigation into the effects of fine coal gangue content, mass concentration, and geopolymer gel binder content on the bearing mechanism of coal gangue geopolymer gel backfill–rock combination (CGBRC) under simulated surrounding rock conditions. To address these research gaps, this study investigates the compressive behavior of coal gangue geopolymer gel backfill–rock combinations (CGBRC) under simulated surrounding rock conditions. Coal gangue is utilized as a full replacement for natural aggregates in geopolymer gel backfill materials, thereby promoting high-value utilization of solid waste and supporting low-carbon green mining practices. Uniaxial compression tests combined with Digital Image Correlation (DIC) and acoustic emission (AE) monitoring are conducted to evaluate the effects of fine gangue content, mass concentration, and binder content on the mechanical properties, strain evolution, crack propagation, and failure characteristics of CGBRC. The findings provide new insights into the cooperative bearing mechanism and damage evolution of geopolymer backfill–rock systems and offer theoretical guidance for coal gangue resource utilization and sustainable mine construction.

## 2. Results and Discussion

### 2.1. Uniaxial Compressive Behaviors of CGBRC

#### 2.1.1. Uniaxial Compressive Strength and Elastic Modulus

**Figure 1:**
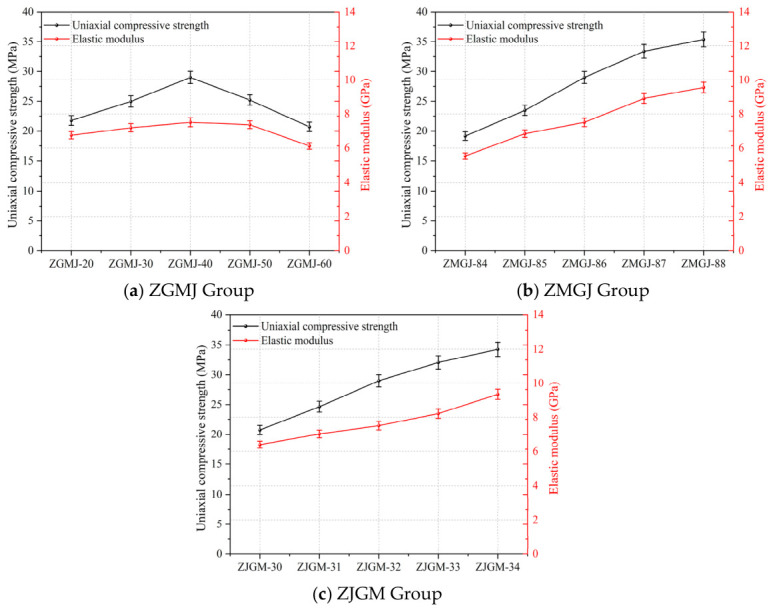
The compressive strength and elastic modulus of the different groups.

The mechanical properties of composites exhibit significant variations with changes in backfill material composition parameters. As illustrated in Figure 1, the uniaxial compressive strength and elastic modulus of CGBRC demonstrate distinct response patterns to different fine gangue content, mass concentration, and binder content.

Figure 1a reveals that both compressive strength and elastic modulus are initially increased and then decreased as the fine gangue content is raised from 20% to 60%, with a peak being reached at a fine gangue content of 40% (ZGMJ-40). At this optimal composition, compressive strength is increased by 39.6% relative to specimens containing 60% fine gangue, while the elastic modulus is increased by 11.2% relative to specimens containing 20% fine gangue. This nonlinear relationship indicates that an optimal balance between particle packing density and binding efficiency is provided by a moderate fine gangue content of 40%, whereas mechanical performance is adversely affected by either excessive or insufficient fine gangue content due to poor gradation or deficient binder distribution.

Figure 1b demonstrates that mechanical properties are continuously enhanced as the mass concentration is increased from 84% to 88%. At a concentration of 88%, compressive strength is increased by 22% and the elastic modulus is increased by 72.8% compared to the 84% concentration specimens. However, practical considerations indicate that the maximum mass concentration should be limited to 86%, as operability is significantly reduced and construction complexity is increased at higher concentrations, while the marginal increase in strength is insufficient to offset the substantial rise in material costs [19].

As shown in Figure 1c, mechanical properties are progressively improved as the binder content is increased from 30% to 34%, with compressive strength being increased by 18.2% and the elastic modulus being increased by 46.3% at a binder content of 34% compared to the 30% reference. However, the enhancement rate is significantly diminished beyond a binder content of 32%, establishing this level as the cost-performance optimum, where strength requirements are satisfied with compressive strength greater than 25 MPa and economic feasibility is achieved without compromising flow characteristics.

The findings are found to be consistent with previous research conducted by Chang et al. [14] and Zhu et al. [20], confirming that the load-bearing capacity of CGBRC is directly correlated with the strength of the backfill material. The optimal composition is established by the parametric study as 40% fine gangue content, 86% mass concentration, and 32% binder content, with balanced mechanical performance achieved while practical applicability is maintained.

#### 2.1.2. Uniaxial Compression Stress–Strain Curve

**Figure 2:**
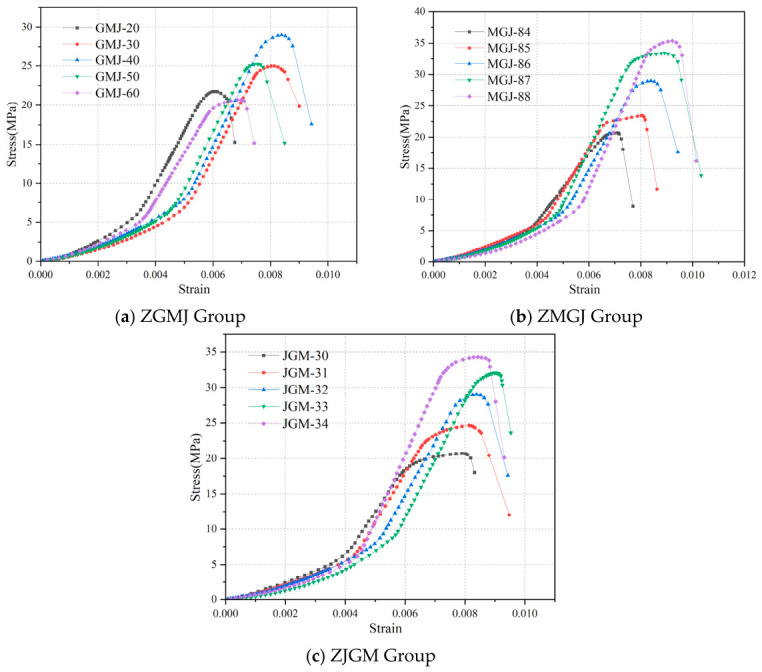
Stress strain curve of CGBRC under different gangue ratios, mass concentrations, and binder contents.

The stress–strain behavior of CGBRC is regarded as a critical indicator for assessing mechanical performance and failure characteristics, with direct implications for engineering bearing capacity and structural stability. Through systematic uniaxial compression testing, comprehensive load-deformation data were obtained, and representative stress–strain curves for the brittle composites were derived, as presented in Figure 2.

Figure 2a demonstrates the pronounced influence of fine gangue content on the stress–strain behavior of CGBRC. Optimal performance is achieved with a 40% fine gangue content formulation, achieving the highest peak stress of 29 MPa and peak strain of 8.64%, which are 15 to 40% and 23 to 35% higher than those of the other groups, respectively. This enhancement is attributed to improved particle packing efficiency. Lower fine gangue contents of 20 to 30% lead to insufficient void filling and decreased compactness, while higher contents of 50 to 60% weaken the skeletal framework formed by coarse aggregates. The 40% fine gangue content provides sufficient fines for matrix densification while maintaining adequate coarse aggregate content for structural support.

As shown in Figure 2b, mass concentration has a significant impact on the deformation behavior of CGBRC. Increasing the concentration from 84% to 88% produces two main effects. The yield strength is increased by up to 84% due to the reduced porosity in the hardened matrix, and ductility is improved, as reflected by an extension of plastic deformation before failure of up to 31%. The improvements are attributed to the increased density and refined pore structure achieved at higher solid content.

As shown in Figure 2c, complex effects on the mechanical behavior of CGBRC are exerted by binder content. Peak stress is consistently increased across the content range of 30% to 34%, while peak strain is first increased and then decreased, reaching a maximum at 33% binder content. This behavior is caused by competing mechanisms. At lower dosages of 30% to 32%, geopolymer gel formation is restricted by the limited availability of reactive aluminosilicate precursors, resulting in a reduction in compressive strength. At higher dosages of 34%, inert “hard cores” are produced by incomplete hydration of excess cementitious material, acting as stress concentrators. Microcracks are initiated, reducing ductility by 2.4% and weakening interfacial bonding through the formation of weak transition zones in the matrix microstructure [21]. At the same time, the peak strain of 8.64% observed at 34% bander content is considered to reflect the best balance between full gel formation and the smallest unreacted particles.

### 2.2. Strain Field Evolution Process

**Figure 3:**
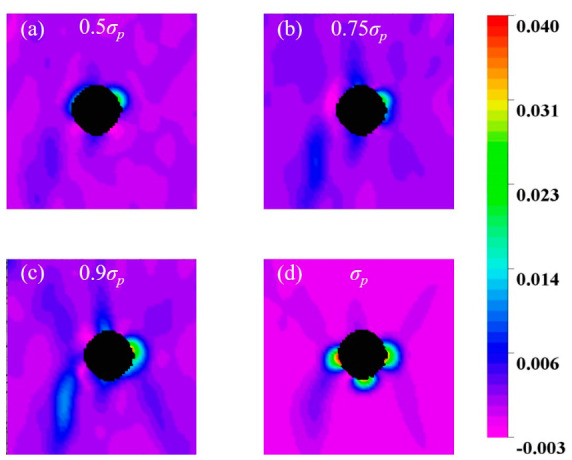
Maximum principal strain field evolution of the pre-drilled hole specimens: (**a**) 0.5 *σ_p_*; (**b**) 0.75 *σ_p_*; (**c**) 0.9 *σ_p_*; (**d**) *σ_p_*.

**Figure 4:**
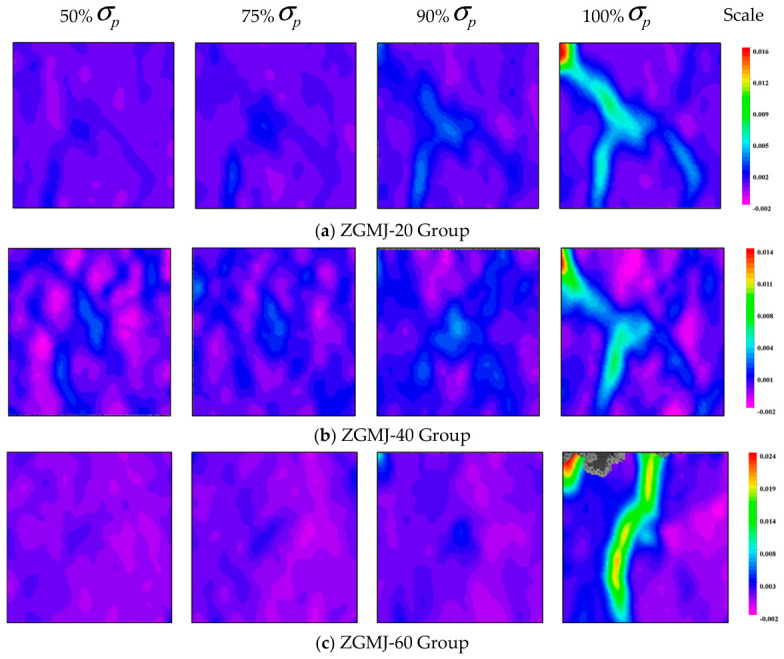
Maximum principal strain field evolution of different contents of fine gangue: (**a**) ZGMJ-20 Group; (**b**) ZGMJ-40 Group; (**c**) ZGMJ-60 Group.

**Figure 5:**
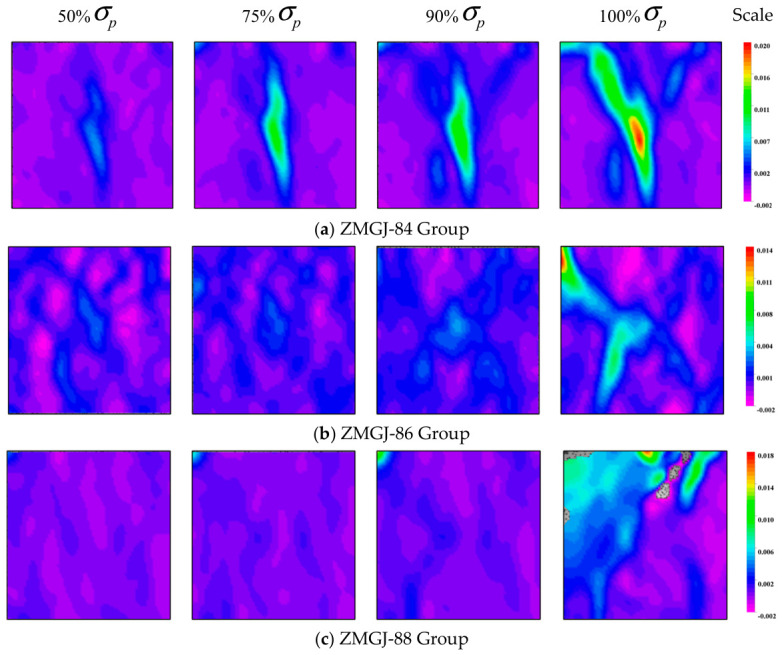
Evolution cloud map of the maximum principal strain of different mass concentrations: (**a**) ZMGJ-84 Group; (**b**) ZMGJ-86 Group; (**c**) ZMGJ-88 Group.

**Figure 6:**
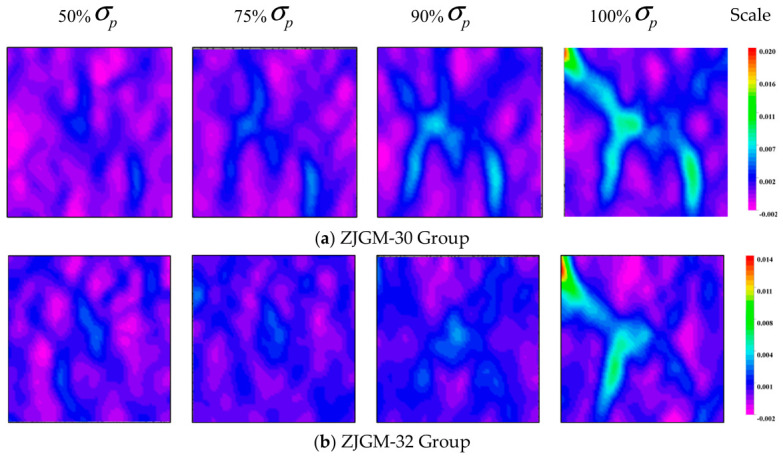
Maximum principal strain field evolution of different binder contents: (**a**) ZJGM-30 Group; (**b**) ZJGM-32 Group.

In this study, the DIC technique was employed to analyze strain fields based on speckle images captured by high-speed cameras during uniaxial compression tests [22]. As illustrated in Figure 3, Figure 4, Figure 5 and Figure 6, maximum principal strain evolution contours were obtained for both pre-drilled specimens and composite specimens with varying compositions at four characteristic stress levels, 50%, 75%, 90% and 100% of peak stress. To simulate the actual stress environment of underground backfill mining, pre-drilled rock-like specimens were used in this study. A cylindrical hole was prefabricated in the center of the rock-like specimen, into which the coal gangue geopolymer backfill slurry was poured and cured to form CGBRC. This design enabled the investigation of the mechanical interaction, strain localization, and failure mechanism between the geopolymer backfill and the surrounding rock matrix under uniaxial compression, providing more realistic mechanical behavior data for engineering applications. Meanwhile, the use of pre-drilled specimens allowed for a direct comparison with pure backfill specimens. By optimizing the color scale calibration, strain concentration zones on specimen surfaces were effectively highlighted, and image contrast and feature discernibility were significantly enhanced. It should be noted that, due to the typical brittle fracture behavior of CGBRC, sudden catastrophic failure accompanied by explosive noises was experienced by the specimens upon reaching peak stress. Therefore, post-peak crack propagation was excluded from analysis, as through-going macroscopic cracks had already formed and structural integrity had been completely lost at this stage.

Figure 3 illustrates the evolution of maximum principal strain fields in pre-drilled specimens. Under initial loading conditions, strain concentration zones were first developed near the pre-drilled hole region. As loading was increased, two prominent strain concentration zones emerged along the upper and lower edges of the hole, while additional strain localization was observed at the lower-left corner of the specimens. When the peak load was approached, significant strain concentration was developed at both the upper and lower corners of the specimens. With continued uniaxial compression, the strain concentration zones near the hole were progressively extended toward the cavity. Distinct shear deformation bands were gradually formed between the high-strain regions and the pre-drilled area. The shear deformation bands were ultimately evolved into macroscopic failure planes intersecting with the hole. Complete failure of the specimens was eventually induced [9].

As shown in Figure 4, Figure 5 and Figure 6, fundamentally different crack initiation and propagation mechanisms are exhibited by CGBRC specimens when compared with pre-holed specimens [7,23]. The interfacial zone between the backfill material and the rock matrix is regarded as the primary weakness, where localized strain concentration is likely to trigger interfacial shear-slip cracks [24,25,26,27]. The interfacial behavior is strongly influenced by the mechanical properties of the backfill material. For lower-strength backfill material such as Figure 4c and Figure 5a, through-going crack penetration is permitted, whereas in higher-strength composites such as Figure 4a, only localized debonding is developed while the integrity of the backfill material is maintained.

Figure 4 presents the evolution of the maximum principal strain field for the ZGMJ-20, ZGMJ-40, and ZGMJ-60 groups at stress levels from 50%σ_p_ to 100%σ_p_. In Figure 4a, the strain field remains relatively uniform at 50%σ_p_ and 75%σ_p_, develops a weak local concentration at 90%σ_p_, and forms a distinct connected shear band with a peak strain of ~0.016 at 100%σ_p_. In Figure 4b, strain distribution is homogeneous at low stress levels, shows scattered concentration at 90%σ_p_, and forms a continuous failure band with a peak strain of ~0.014 at 100%σ_p_, exhibiting a more diffuse damage pattern than Figure 4a. In Figure 4c, the strain field remains nearly uniform up to 75%σ_p_ with only minor anomalies at 90%σ_p_, and at 100%σ_p_, a significantly wider and more intense strain band emerges with a peak strain reaching 0.024, indicating a more concentrated and severe failure process.

Figure 5 shows the evolution of the maximum principal strain field for the ZMGJ-84, ZMGJ-86, and ZMGJ-88 groups under stress levels from 50%σ_p_ to 100%σ_p_. In Figure 5a, a clear strain band begins to form as early as 50%σ_p_, intensifies and extends continuously through subsequent loading stages, and finally develops into a fully connected, concentrated failure zone with a peak strain of approximately 0.020 at 100%σ_p_. In Figure 5b, strain distribution remains relatively uniform at low stress levels, with scattered strain concentration appearing at 90%σ_p_, and a distinct cross-shaped failure band forming at 100%σ_p_ with a peak strain of about 0.014, reflecting a more diffuse damage evolution compared to Figure 5a. In contrast, Figure 5c shows a nearly homogeneous strain distribution up to 90%σ_p_, with no obvious localized concentration, and at 100%σ_p_, a wide and irregular strain zone rapidly emerges, with the peak strain reaching approximately 0.018, indicating an abrupt and unstable failure process at the final loading stage.

Figure 6 illustrates the evolution of the maximum principal strain field for the ZJGM-30 and ZJGM-32 groups at stress levels from 50%σ_p_ to 100%σ_p_. In Figure 6a, an inclined strain band begins to form at 50%σ_p_, gradually intensifies and extends at 75%σ_p_ and 90%σ_p_, and ultimately develops into a fully connected, highly concentrated failure zone at 100%σ_p_ with a peak strain of approximately 0.020, showing a progressive and localized damage evolution process. In Figure 6b, the strain field remains relatively homogeneous at low stress levels, with only scattered and weak strain fluctuations appearing at 50%σ_p_, 75%σ_p_, and 90%σ_p_; at 100%σ_p_, a distinct cross-shaped strain band suddenly emerges with a peak strain of about 0.014, reflecting a more diffuse and delayed failure process compared to Figure 6a.

Overall, the strain field evolution process of CGBRC specimens is characterized by three stages. First, strain concentration is observed at the tips of the backfill material under loading. Second, high-strain localization zones are developed. Third, secondary cracks are initiated, and the propagation patterns are directly governed by the strength of the backfill material. Shear-dominant crack initiation is promoted by enhanced mechanical properties [23,28,29]. End effects are manifested through anti-symmetric propagation from the backfill material tips toward the specimen corners. Characteristic X-shaped shear or hybrid tensile-shear failure surfaces are ultimately formed. The results indicate that the failure mechanics of CGBRC are governed by two competing factors. Initial damage localization is determined by interfacial bonding strength, while subsequent crack evolution pathways are controlled by the bulk mechanical properties of the backfill material. The transition from interfacial debonding to matrix penetration and finally to system-scale failure illustrates a strength-dependent hierarchical damage accumulation process. Critical criteria for performance-based material design in underground engineering applications are thus provided.

### 2.3. Damage Process of CGBRC Based on AE Cumulative Count

**Figure 7:**
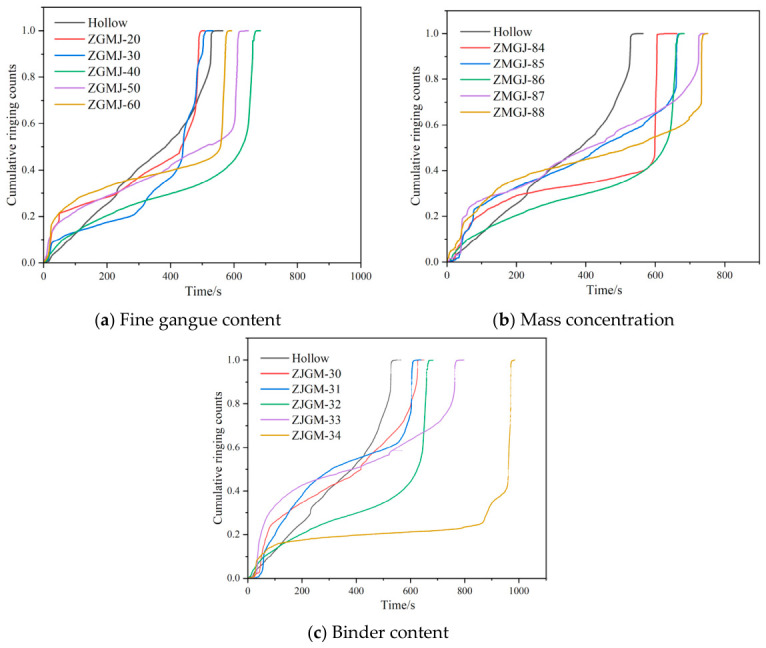
Relationship between cumulative AE count and time under different gangue rates, mass concentrations, and binder contents of CGBRC.

Through AE analysis with normalized processing of CGBRC specimens with different compositions, the relationship between time and cumulative ringing counts was established, as shown in Figure 7. Damage evolution is characterized by three distinct phases. In the early compaction stage, AE signals are rapidly accumulated, while ringing counts exhibit minor fluctuations under external loading and remain relatively low, accounting for only a small portion of total cumulative counts [30]. The behavior is caused by pore compaction under pressure without aggregate fracture, and limited AE signals are generated primarily by cracking [31]. After initial compaction, a relatively stable and progressive phase is entered, during which the accumulation rate is significantly slowed. Once the CGBRC specimens enters the yield stage, a marked surge in cumulative AE counts is observed, accompanied by considerable fluctuations throughout the test [32,33]. Overall, the influence of backfill material composition on internal structure evolution of the CGBRC is prominently demonstrated during the compaction and elastic stages.

Figure 7a presents the normalized evolution curves of cumulative ringing counts of CGBRC with varying fine gangue content from 20% to 60%. During the compaction stage, cumulative counts are initially decreased and then increased as fine gangue content rises. Substantial AE activity is observed in specimens with 20% fine gangue content during the initial loading phase due to particle rearrangement. When fine gangue content reaches 40%, particle packing density is improved, with voids between coarse aggregates effectively filled by fine particles, and crack propagation pathways are significantly restricted, resulting in markedly reduced AE activity. Beyond the optimal 40% threshold, the presence of excessive fine particles increases the specific surface area, leading to greater absorption of free water and higher slurry viscosity. During hardening, differential shrinkage between fine particles generates microvoids, which elevate interparticle friction and enhance AE activity under loading. The process explains the resurgence in cumulative counts observed at higher fine gangue content.

Figure 7b shows the normalized evolution curves of cumulative ringing counts for CGBRC with varying mass concentrations from 84% to 88%. During the compaction stage, cumulative counts first increase, then decrease, and finally increase again as mass concentration rises. At a mass concentration of 84%, significant AE activity is produced in the early stage due to relatively poor material compactness. At a mass concentration of 86%, a sharp increase is observed only at the point of failure. At that concentration, structural stability and strength are maximized, and the risk of early damage caused by excessive brittleness is avoided. CGBRC stability is significantly increased as compactness is improved, particle spacing is reduced, and structural stability along with cooperative load-bearing capacity is enhanced [32,33]. At a mass concentration of 88%, the CGBRC specimens becomes excessively dense and brittle, workability is dramatically reduced, and sudden damage and rapid propagation at the initial loading stage are promoted, resulting in a sharp early surge in ringing counts.

Figure 7c presents the normalized evolution curves of cumulative ringing counts of CGBRC with different binder content from 30% to 34%. During the compaction stage, the cumulative counts show an initial rise, followed by a decline, and then a subsequent increase as the binder content increases. With 30% binder content, pronounced AE activity is observed during the initial loading stage. At 32% binder content, particles are fully encapsulated by the enhanced cementation effect, and a denser overall structure is formed, effectively suppressing particle friction and microcrack propagation during loading, resulting in a significant and sustained reduction in cumulative AE ringing counts. When the binder content reaches 34%, incomplete reactions and larger pores are observed. The internal weaknesses are created by insufficient hydration and uniformity issues caused by excessive binder content, and potential sources of early damage risk are introduced.

The sudden surge in AE ringing counts is detected as an early warning indicator of approaching critical damage, and valuable insight is provided for predicting damage evolution [34,35]. The stages of damage initiation, propagation, and accumulation during loading are revealed by AE ringing count analysis of CGBRC, and essential support is offered for understanding progressive damage and failure mechanisms.

**Figure 8:**
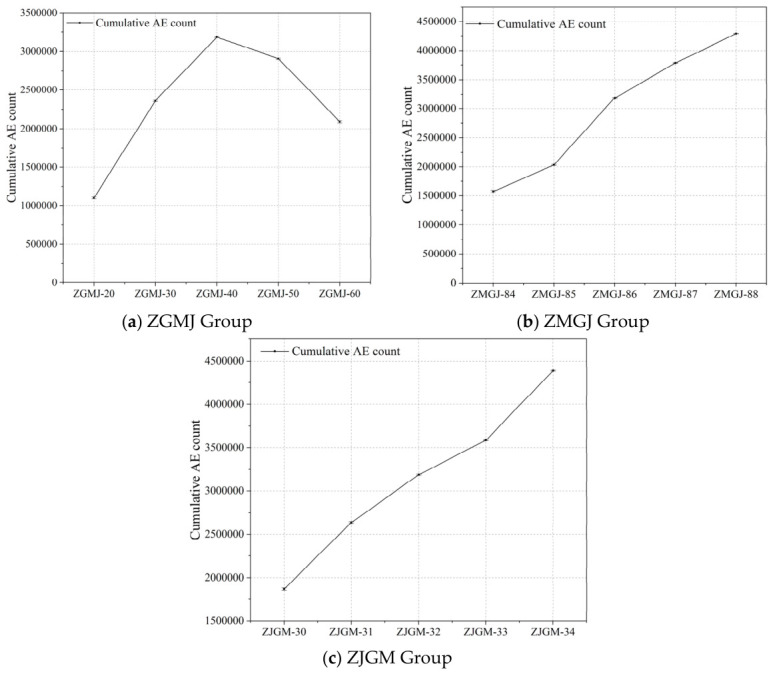
Accumulated ringing count of CGBRC under different gangue rates, mass concentrations, and cementitious material contents.

Complementing the observations, Figure 8a demonstrates that total cumulative ringing counts follow a unimodal trend, peaking at 40% fine gangue content. Maximum AE activity is likely associated with the densest microstructure of the material, where a high frequency of AE events is generated by the sudden release of accumulated strain energy during brittle failure, producing the observed peak in total counts. As shown in Figure 8b,c, an increase in cumulative ringing counts is observed with both mass concentration and binder content, consistent with the trend observed in compressive strength of CGBRC.

### 2.4. Failure Mode of CGBRC

**Figure 9:**
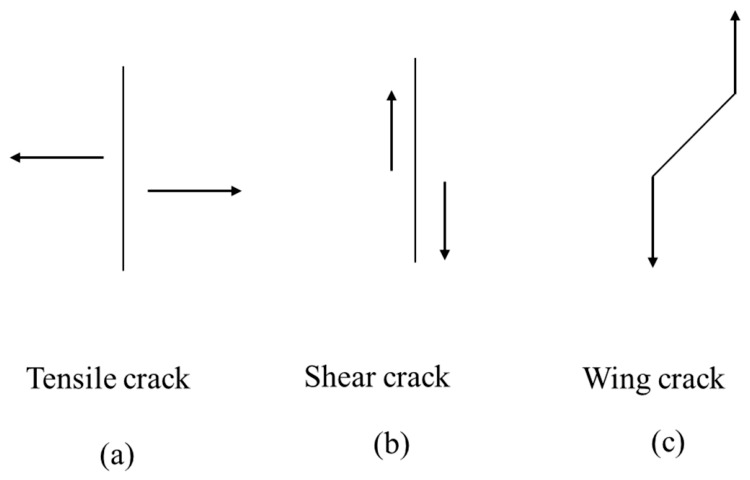
Typical crack type diagram: (**a**) tensile crack; (**b**) shear crack; (**c**) wing crack.

**Figure 10:**
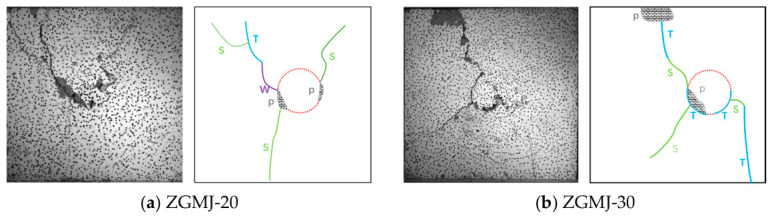

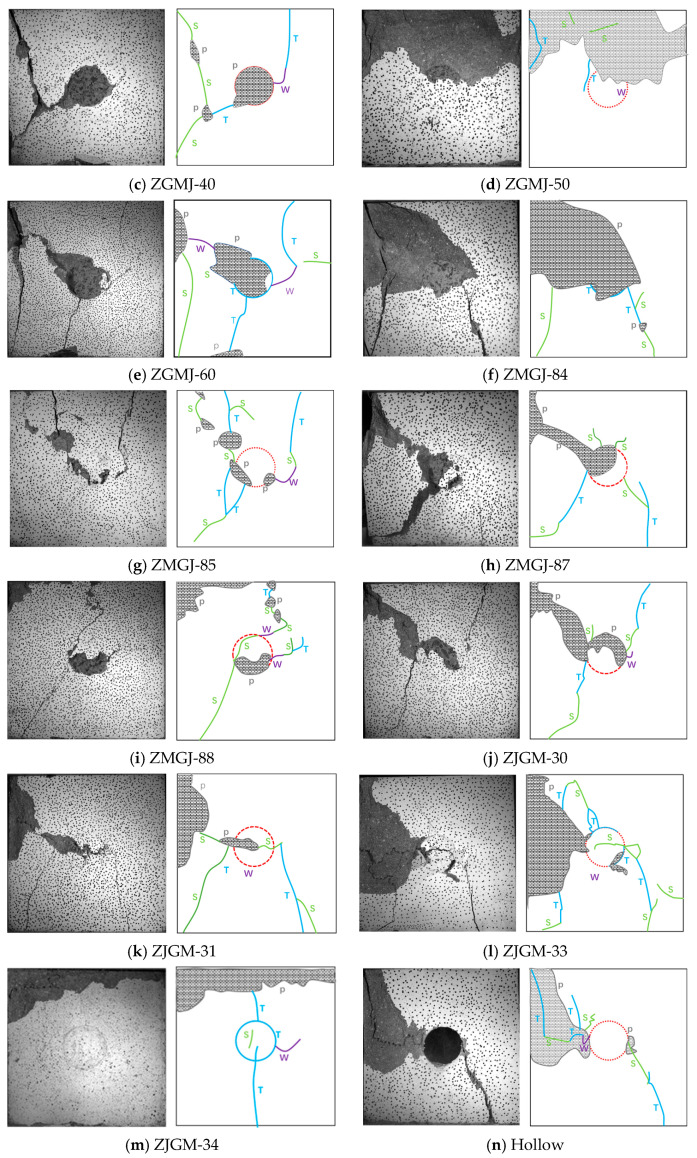
Crack types of CGBRC under different gangue ratios, mass concentrations, and binder contents.

The failure process of CGBRC involves multiple crack types, which are classified based on displacement trendlines. Typical wing cracks, shear cracks, and tensile cracks are shown in Figure 9 [36,37]. The failure patterns and crack distributions of various specimen types are presented in Figure 10, with spalling exhibited to varying degrees and the most extensive spalling zones observed in the unfilled CGBRC specimens. While failure modes remain generally similar across different backfill material compositions, all specimens are characterized by brittle fracture under compression, resulting in a loss of structural integrity [38].

The specimens exhibited characteristic crack propagation patterns under axial loading. Primary cracks were initiated from both lateral ends of the backfill material and extended towards the top and bottom of the specimens. Concurrently, tensile cracks were developed from the upper and lower ends of the backfill material and propagated along the loading direction, as shown in Figure 10. The distinct crack distribution is governed by the stress distribution within the specimens, with compressive stress concentrated at the lateral ends of the backfill material and tensile stress concentrated at the upper and lower ends. In addition, two distinct crack interaction pathways were observed around the backfill material, with some cracks connected along the interface and others penetrating through the backfill material. The bifurcation behavior is determined by the mechanical properties of CGBRC. The spatial variation in crack coalescence paths highlights the anisotropic response of the material to compressive stresses and reveals significant heterogeneity in its failure mechanisms.

The interfacial failure behavior of CGBRC is primarily governed by the strength disparity between the backfill material and surrounding rock matrix. Three characteristic failure modes are revealed by analysis of crack propagation paths based on their geometric interaction with the backfill material. Type I, penetrating backfill material failure, the predominant mode, is observed when cracks fracture through the geopolymer gel matrix, which is specifically manifested by fragmentation of the backfill material as shown in Figure 10c,f,j. Type II, axisymmetric interfacial failure, is observed when cracks are initiated from critical endpoints of the backfill material and propagated vertically, which causes partial interface spalling with 25–50% interfacial debonding as shown in Figure 10h,l. Type III, centrosymmetric interfacial failure, is characterized by cracks symmetrically distributed along the interface, which produces extensive interfacial debonding of 50–100% as shown in Figure 10i,m. Initial damage initiation is governed by interfacial weakness, while the ultimate failure mechanism is transitioned from interfacial debonding to matrix penetration as the backfill material-to-rock strength ratio decreases. In addition, some backfill material had already failed without obvious spalling due to automatic failure detection by the testing machine, as illustrated in Figure 10a,b.

Table 1 summarizes the failure modes of different specimens. With the fine gangue content increased from 20% to 60%, the failure mode was consistently maintained as Type I. The compressive strength of the backfill material was not significantly altered by variations in fine aggregate content within this range and remained lower than the matrix strength. Consequently, penetrating backfill material failure of Type I was observed in the specimens.

The mass concentration of CGBRC was also found to notably influence the failure mechanism. With the mass concentration increased from 84% to 88%, the failure mode was observed to transition from Type I to Type II and eventually to Type III. The transition was primarily attributed to the increase in strength with higher mass concentration, which caused the stiffness ratio to gradually approach 1 from a value far less than 1, thereby promoting Type III failure. On the other hand, sufficient penetration and uniform coating at local interface areas may have been hindered by a sharp decline in slurry fluidity, leading to inadequate gel structure formation, reduced interfacial bond strength, and ultimately extensive interface failure.

Binder content also significantly influenced the failure mode. At low binder contents of 30–32%, Type I failure was induced. At this level, the total amount of hydration products was insufficient to effectively bind the particles, leaving the strength of the backfill material markedly lower than that of the surrounding rock matrix. Consequently, the backfill material became the weakest link, and penetrating failure was triggered. At moderate binder content of 33%, Type II failure was exhibited. Stress concentration near the interface was generated by the enhanced strength of backfill material, inducing partial interfacial debonding, followed by crack propagation into the matrix, which led to specimen failure. At a high binder content of 34%, Type III failure was observed. At this level, the strength of the backfill material matched that of the matrix, which caused stress concentration at the interface and promoting extensive debonding. Additionally, defects at the interface were created by unhydrated particles introduced by excessive binder, further contributing to interfacial failure.

### 2.5. Influence Mechanism of Backfill Composition on the Strength of CGBRC

**Figure 11:**
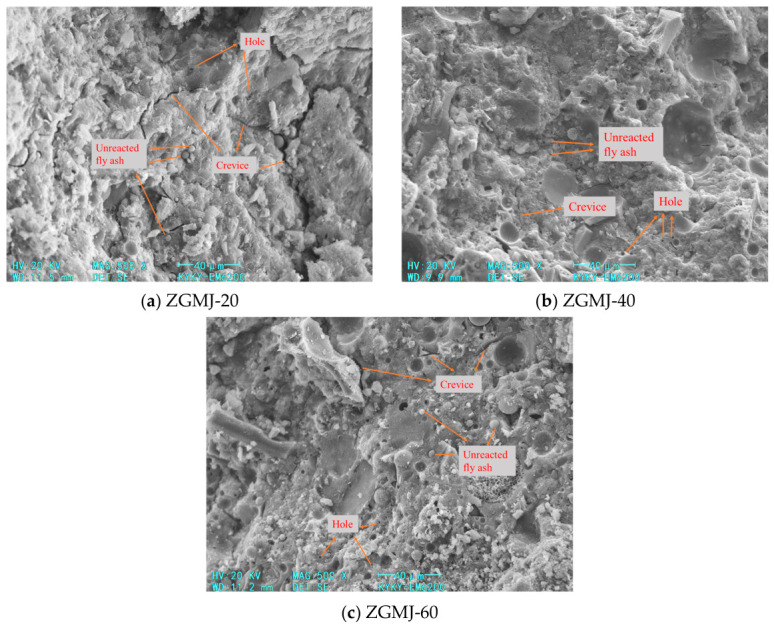
SEM images of backfill material with different fine gangue contents.

The mechanical properties of CGBRC are significantly influenced by backfill material composition. At a fine gangue content of 40%, both the compressive strength and elastic modulus of CGBRC are enhanced through improving the density of backfill material, as confirmed by SEM analysis shown in Figure 11. When fine gangue content is increased from 20% to 40%, the microstructure becomes more homogeneous, and fine particles are effectively encapsulated by geopolymer gel, which reduces pores and cracks as shown in Figure 11b. This densification is achieved through improved particle packing and enhanced geopolymerization, in which abundant amorphous gel is generated by alkaline-activated fly ash and slag to fill voids and strengthen the CGBRC [39]. At an excessive fine gangue content of 60%, the specific surface area is increased, free water is adsorbed, and microcracking is promoted by interparticle friction, ultimately resulting in degraded mechanical performance [40].

**Figure 12:**
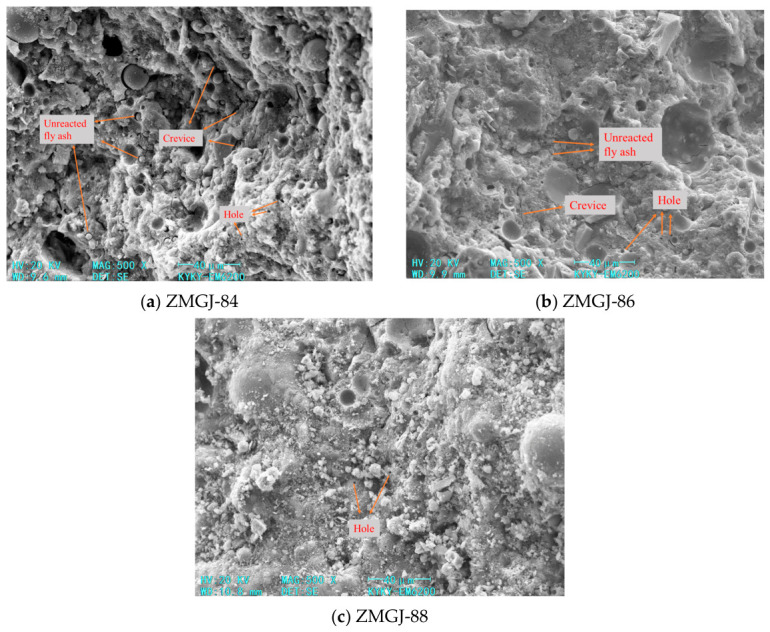
SEM images of backfill material with different mass concentrations.

The mechanical performance of CGBRC is strongly influenced by mass concentration through its effect on microstructural evolution. Under low-concentration conditions, excess free water is retained and interconnected porous networks are formed during the hardening process, which results in stress concentration zones that facilitate microcrack propagation and lead to strength reduction, as shown in Figure 12a [39]. At higher mass concentrations, more favorable microstructural characteristics are developed, in which increased geopolymer gel formation fills pores of various sizes and the amount of unreacted particulate matter is minimized, as shown in Figure 12b,c. With reduced free water content, concentrated geopolymer gels completely seal microscopic voids, which results in a dense matrix with superior structural integrity and load transfer capability. The microscopic mechanism is confirmed to align with earlier findings regarding the enhancement of elastic modulus [40,41]. In addition, the geopolymerization process primarily produces N-A-S-H and C-A-S-H gel phases, which dominate the compressive and flexural strengths of the backfill material. The N-A-S-H gel constructs a stable three-dimensional network and improves the compactness of the matrix. Meanwhile, the C-A-S-H gel strengthens the interfacial bonding between aggregates and the binder matrix, thus greatly boosting the compressive strength [5,42]. The water-to-solid ratio determines the formation, cross-linking degree, and microstructure of these gels, and further controls mortar consistency and strength development. A low water-to-solid ratio reduces internal porosity and significantly improves compressive strength. However, an extremely low water-to-solid ratio increases interparticle friction, limits plastic deformation, and weakens energy dissipation during crack propagation, resulting in reduced ductility and more brittle flexural failure [41]. These findings are consistent with the latest advances in high-strength geopolymer concrete, which indicate that optimized precursor composition, alkaline activator ratio, and water-to-solid ratio are critical to obtaining a dense gel structure and excellent mechanical properties for sustainable construction [43].

**Figure 13:**
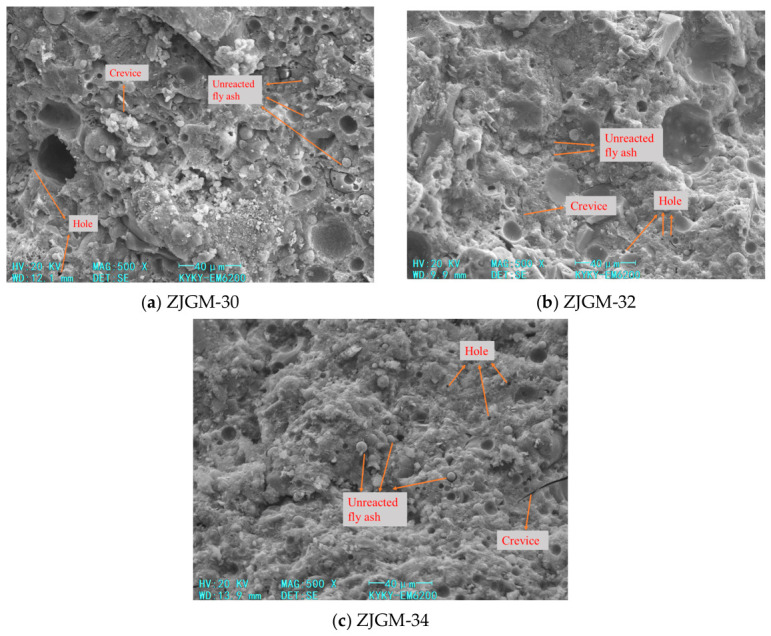
SEM images of backfill material under different binder contents.

The mechanical properties of CGBRC demonstrate a consistent enhancement with increasing binder content from 30% to 34%. At a lower binder content of 30%, numerous unreacted spherical FA particles remain dispersed throughout the matrix, which indicates insufficient geopolymerization that results in sparse and unevenly distributed gel formation along with substantial porosity, as illustrated in Figure 13. The loose microstructure exhibits limited load-bearing capacity, which leads to reduced compressive strength and elastic modulus. Progressive increases in binder content produce microstructural improvements, as shown in Figure 13b,c. The matrix surface becomes progressively smoother with fewer voids, while the increased production of geopolymer gel effectively fills gaps between aggregates, which densifies the overall structure. This microstructural optimization stems from two synergistic mechanisms. First, the abundant availability of reactive silicon and aluminum components facilitates thorough geopolymerization, which generates substantial amorphous aluminosilicate gel that tightly binds particles and strengthens interfacial connections. Second, the increased silicate ions participate more fully in polymerization reactions, which produces additional gelling material that fills and seals microvoids within the specimens [44]. The resulting dense microstructure features large particles forming a skeletal framework, while finer particles occupy interstitial spaces [45]. Such an arrangement creates a stable internal architecture that simultaneously improves compressive strength through enhanced particle bonding and elevates elastic modulus by effectively restraining elastic deformation under external loads. Observed macroscopic property enhancements across the tested binder content range are explained by coordinated microstructural developments [46].

**Figure 14:**
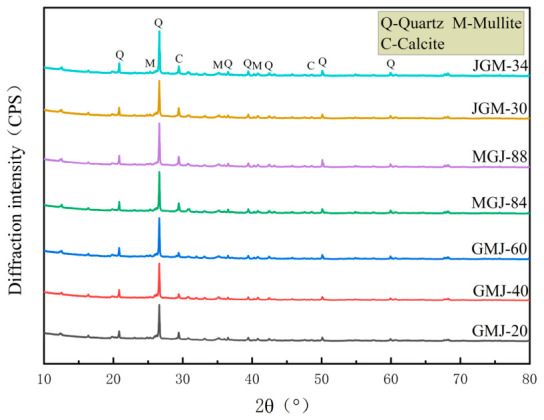
XRD parrerns of backfill material.

The XRD patterns of representative coal gangue geopolymer backfill samples are presented in Figure 14. As shown, all samples exhibit distinct diffraction peaks corresponding to quartz (Q), mullite (M), and calcite (C), which originate from the unreacted coal gangue and fly ash precursors. These crystalline phases remain stable during the alkali activation process and act as inert fillers in the geopolymer matrix. A broad diffraction hump in the range of 2θ = 20–35° is observed in all samples, which is characteristic of amorphous aluminosilicate gel formed by geopolymerization. With the increase in binder content and mass concentration, the intensity of the crystalline peaks slightly decreases, while the broad hump becomes more pronounced, indicating an enhanced degree of geopolymerization and increased formation of amorphous gel products. The presence of residual crystalline phases and the development of the amorphous hump confirm that the improvement in mechanical properties is closely related to the formation of geopolymer gel and the densification of the microstructure.

### 2.6. Interfacial Debonding Mechanism Between Backfill Material and Matrix

**Figure 15:**
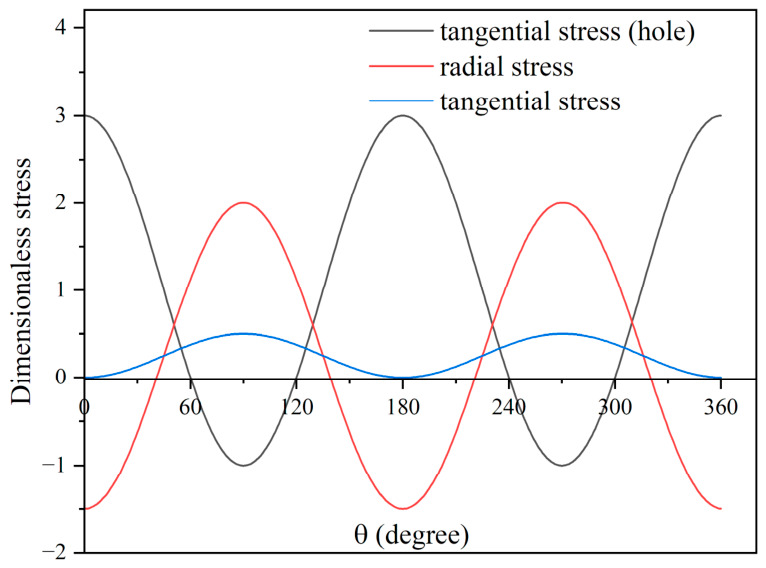
Dimensionless stress concentration factors for a circular hole and a rigid inclusion.

The failure modes of specimens are intrinsically linked to the stress distribution around the backfill material, which makes stress field analysis crucial for experimental interpretation. Under far-field loading, the applied stress is decomposed into tangential and radial components, which allows for the derivation of dimensionless stress coefficients. As illustrated in Figure 15, unfilled specimens are characterized by a tangential stress coefficient of 3 along lateral boundaries but −1 at vertical boundaries, which correlates with observed shear crack initiation at both ends and subsequent structural failure. With the introduction of backfill material, this stress regime is fundamentally altered. Assuming an infinite Young’s modulus for the backfill material, the tangential coefficient is reduced to 0.5 at the top and bottom and to 0 at the left and right, while the radial coefficient reaches −1.5, which produces interfacial tensile conditions that promote debonding between the backfill material and surrounding rock matrix [47,48]. The transition from shear-dominated failure in unfilled specimens to interfacial debonding in the CGBRC is accounted for by stress redistribution.

However, the damage caused by different backfill materials remains difficult to explain in depth, which makes complex variable theory a potential approach for clarifying the underlying reasons for the variations in damage behavior [49]. Tangential stress and radial stress are determined by Equations (1) and (2), respectively.

(1) $$\sigma_{\theta \theta ,j}^{\mathrm{normalized}}=\frac{1}{2}\left\{\left.1+B{\left(\frac{a}{r}\right)}^{2}-\left[1-3C{\left(\frac{a}{r}\right)}^{4}\right]\cos2(\theta +90)\right\}\right.$$

(2) $$\sigma_{rr,i}^{normalized}=\frac{1}{2}\left\{\left.1-B{\left(\frac{a}{r}\right)}^{2}+\left[1-2A{\left(\frac{a}{r}\right)}^{2}-3C{\left(\frac{a}{r}\right)}^{4}\right]\cos2(\theta +90)\right\}\right.$$

where $\sigma_{\theta \theta ,j}^{normalized}$ and $\sigma_{rr,i}^{normalized}$ are the tangential and radial stress concentration coefficient, respectively; *β* is the ratio of inclusion elastic moduli to matrix elastic moduli; *a* is the radius of the filling material; and *r* is the polar coordinates with the center of the filling material as the origin. The parameters *β*, *A*, *B*, and *C* are determined by Equations (3)–(6).

(3) $$\beta =\frac{G_{i}}{G}$$

(4) $$A=\frac{2(1-\beta )}{2\beta +1}$$

(5) $$B=\frac{1-\beta }{2\beta +1}$$

(6) $$C=\frac{\beta -1}{2\beta +1}$$

where *G_i_* and *G* represent the elastic modulus of the backfill material and the CGBRC, respectively. *A*, *B*, and *C* are the coefficients used in the equations.

**Figure 16:**
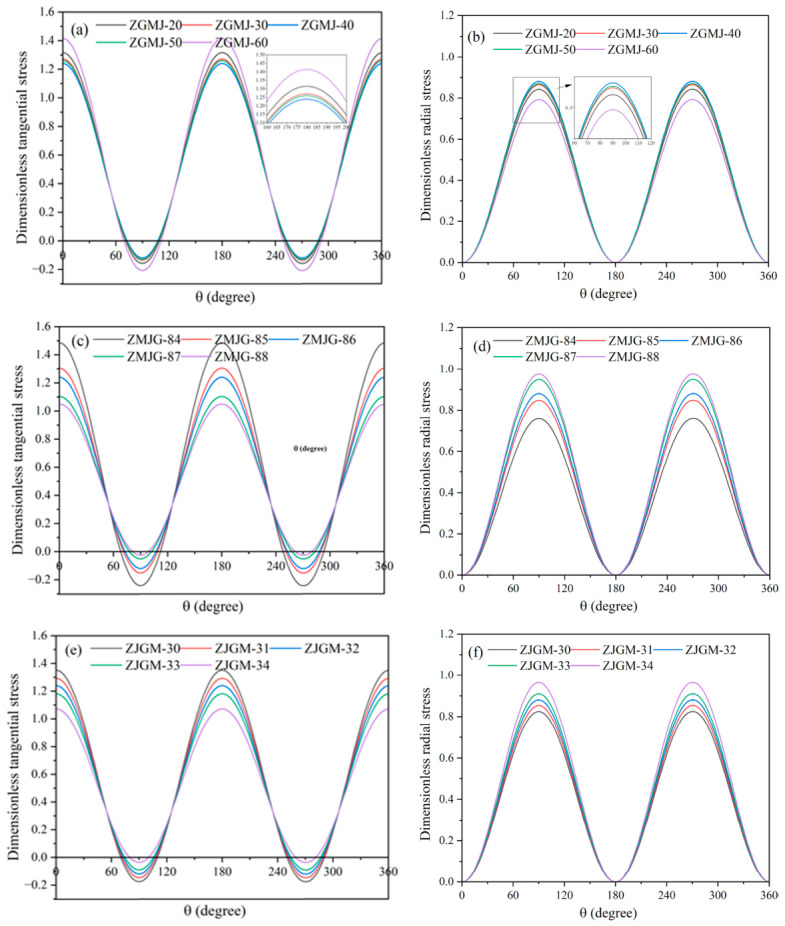
Normalized tangential stress concentration factors: (**a**) ZGMJ group; (**c**) ZMJG group; (**e**) ZJGM group; Normalized radial stress concentration factors: (**b**) ZGMJ group; (**d**) ZMJG group; (**f**) ZJGM group, around the backfill with different materials.

Table 2 presents the compressive strength and elastic modulus of different backfill materials. According to Equation (3), the stiffness ratio of different backfill materials to the matrix is obtained. Figure 16 illustrates the normalized tangential and radial stress concentration factors derived for different backfill materials. When the stiffness ratio β is less than 1, the tangential stress amplification at the transverse edges indicates a compressive stress state, while the negative values at the upper and lower edges indicate a tensile stress state. As the stiffness ratio increases, the tangential tensile stress at the upper and lower edges of the backfill material decreases, indicating a reduced tendency for tensile cracks to initiate in these regions [50]. Meanwhile, the radial compressive stress at that location increases, which results in interface debonding. As the stiffness ratio increases, the tangential compressive stress on both sides of the backfill material decreases, which shows that crack development from the sides becomes more difficult. This corresponds to the observed cracks in Figure 10. In addition, as shown in Figure 14, the radial stress of the inclusion at the 0° position is 0, while the tangential stress at this position is negative, indicating that as the stiffness ratio increases, the tensile stress along the lateral edges of the inclusion gradually rises, enhancing the likelihood of interface debonding, as shown in Figure 10m.

## 3. Conclusions

Uniaxial compression tests were conducted to systematically investigate the effects of fine gangue content (20–60%), mass concentration (84–88%), and binder content (30–34%) on the mechanical properties of coal gangue geopolymer gel backfill–rock combinations (CGBRC). Digital Image Correlation (DIC) and acoustic emission (AE) techniques were employed to characterize the strain field evolution and damage progression. The main conclusions are drawn as follows:

(1) The compressive strength and elastic modulus of CGBRC first increase and then decrease with the increase in fine gangue content, reaching the maximum at 40% fine gangue content. In comparison, the mechanical properties increase continuously with the increase in mass concentration and binder content, but the growth rate slows down significantly after 86% mass concentration and 32% binder content. The optimal mix proportion obtained in this study is 40% fine gangue content, 86% mass concentration, and 32% binder content, under which the compressive strength of CGBRC can reach more than 25 MPa, meeting the long-term bearing and deformation control requirements of mine backfill engineering. The cumulative AE ringing count is consistent with the compressive strength, which can be used as an effective index to characterize the internal damage degree and stability state of CGBRC.
(2) Strain localization of CGBRC first appears near the backfill–rock interface, and the crack propagation path is controlled by the strength of backfill material. The damage evolution experiences three typical stages: rapid damage accumulation in the compaction stage, steady development in the elastoplastic stage, and sudden acceleration at failure. The DIC and AE results show that the backfill–rock interface is the weakest part of the composite structure. For engineering applications, enhancing the interface bonding state and improving the uniformity of backfill materials can effectively delay strain localization and crack initiation.
(3) The interfacial debonding behavior of CGBRC is dominated by the strength mismatch between backfill and surrounding rock. According to the crack propagation path, three failure modes are determined: penetrating backfill failure, axisymmetric interface failure, and centrally symmetric interface failure. With the increase in backfill strength, the failure mode changes from penetrating the internal backfill to interface debonding. For mine backfill engineering, the backfill strength should be matched with the surrounding rock strength to avoid sudden interface debonding and ensure the overall stability of the filling structure.
(4) For practical engineering application, the recommended design strength of coal gangue geopolymer backfill material is not less than 14 MPa, and the optimized mix proportion proposed in this study can fully meet the strength requirements of controlling strata movement, reducing surface subsidence, and ensuring roadway stability. The research results can provide direct parameters and theoretical support for the on-site mixing proportion design, pumping construction, quality control, and safety early warning of coal gangue geopolymer backfill.

## 4. Materials and Methods

### 4.1. Materials

**Figure 17:**
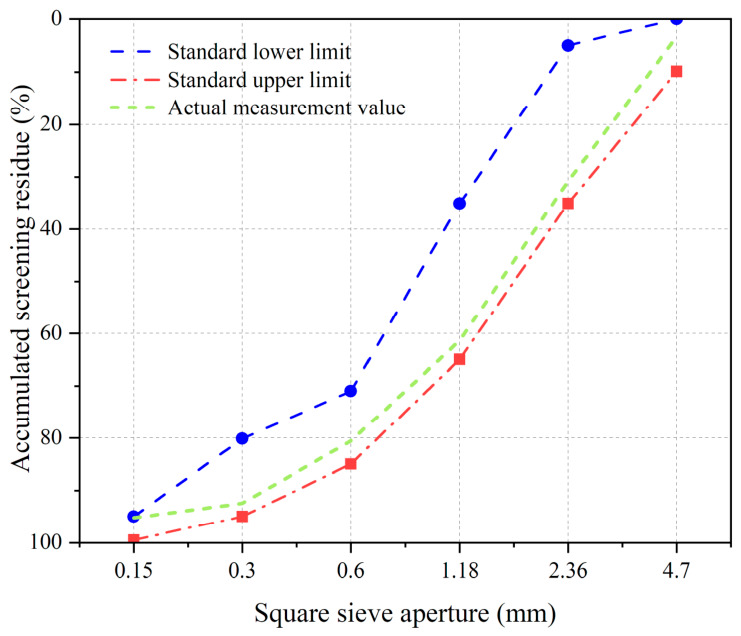
Particle size distribution of fine aggregate.

Fly ash (FA) and ground granulated blast-furnace slag (GGBS) were used together as geopolymer precursors, a NaSiO_3_-NaOH composite solution (modulus 1.3) was used as the alkaline activator, and coal gangue with graded particle sizes (0–5 mm fine aggregate, 5–10 mm and 10–15 mm coarse aggregate) was used as aggregates to prepare CGBRC. The physical parameters and chemical composition of FA and GGBS are presented in Table 3 and Table 4, respectively. Particle size analysis (Figure 17) demonstrated that the gradation curve of coal gangue fine aggregates closely matched that of standard sand, confirming that coal gangue is technically feasible as a complete substitute for river sand.

### 4.2. Specimens Preparation

The influence of three key parameters on mechanical properties was systematically investigated. Fine gangue content, defined as the percentage of fine gangue mass to total gangue mass, is represented by the letter G in Table 5. Mass concentration, defined as the ratio of solid material mass to total slurry mass, is represented by the letter M in Table 5. Binder content, defined as the percentage of binder mass to total solid material mass, is represented by the letter J in Table 5. A five-level experimental design based on the controlled variable method was adopted. Fine gangue content was set at 20%, 30%, 40%, 50%, and 60%, mass concentration was set at 84%, 85%, 86%, 87%, and 88%, and binder content was set at 30%, 31%, 32%, 33%, and 34%. During testing, when one variable was examined, the other parameters were maintained at baseline values of 40% fine gangue content, 86% mass concentration, and 32% binder content. The test groups are named according to the main variable factor in each group: ZGMJ group: The variable is the fine gangue content, with binder content and mass concentration kept constant. ZJGM group: The variable is the binder content, with fine gangue content and mass concentration kept constant. ZMGJ group: The variable is the mass concentration, with fine gangue content and binder content kept constant. Taking the ZGMJ-20 group as an example, the backfill material contains 20% fine gangue, 86% mass concentration, and 32% binder, and is used to prepare the CGBRC. Moreover, unfilled specimens were used as the reference group for comparison.

**Figure 18:**
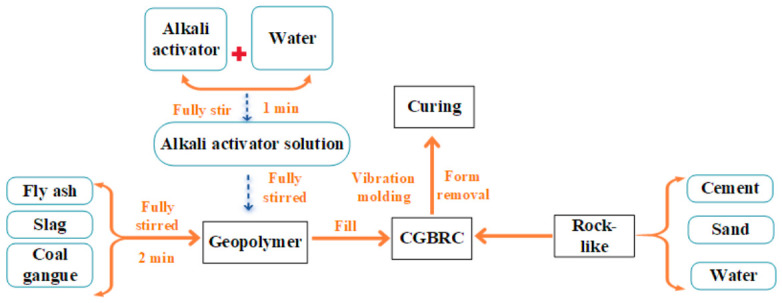
Preparation flowchart of CGBRC.

As shown in Figure 18, rock analog shells are first fabricated using cement mortar in a 2:5:1 ratio of cement, sand, and water, with mechanical properties closely matching natural sandstone to effectively simulate in situ rock behavior [9]. Moreover, the mechanical properties of the prepared rock-like specimens and natural sandstone are compared in Table 6. A Φ75 mm paper-wrapped cylindrical mold core is then positioned in the center of a 300 mm × 300 mm × 60 mm standard formwork and removed after the initial setting of the cement mortar to create standardized cavities. Freshly prepared coal gangue geopolymer gel backfill material, containing a 95:5 fly ash-slag binder system and 1:1 blended 5–10 mm and 10–15 mm graded coal gangue aggregates, is subsequently cast into the cavities. Finally, the specimens are subjected to 24 h of static curing before being demolded and transferred to a standard curing room for subsequent curing.

### 4.3. Methods

**Figure 19:**
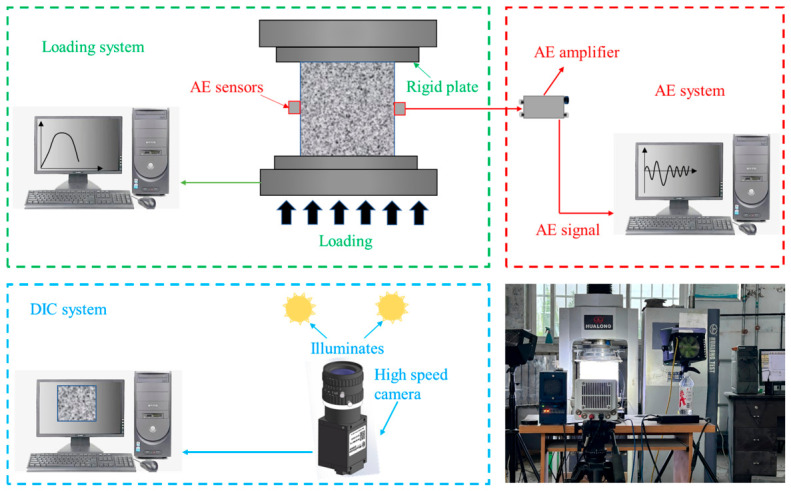
Schematic diagram of uniaxial compression test experimental device.

As shown in Figure 19, the testing system is composed of a WHY-2000 computer-controlled compression machine, a DIC strain measurement system, and an AE monitoring system, enabling the simultaneous acquisition of mechanical properties, full-field surface strain evolution, and internal damage characteristics, and facilitating multiscale analysis linking macroscopic behavior with microscopic failure mechanisms.

#### 4.3.1. Uniaxial Compression Test and Elastic Modulus Test

The uniaxial compressive strength and elastic modulus tests are conducted in accordance with relevant standards [52]. After 28 days of standard curing, uniaxial compression tests were conducted using a WHY-2000 electro-hydraulic servo universal testing machine (Hualong, Shanghai City, China) under load control at a constant rate of 800 N/s until specimen failure occurs. This loading rate was predetermined to avoid dynamic effects while ensuring complete stress–strain curve acquisition. As shown in Table 7, 39 specimens were tested for uniaxial compressive strength, and another 39 specimens were used for elastic modulus tests. The elastic modulus test was then conducted using a WAW-1000 electro-hydraulic servo universal testing machine (Hualong, Shanghai City, China). Specimen deformation was measured by installing micrometer gauges on the specimen surface via a micro-deformation measurement fixture. According to the test specification, three parallel specimens were tested for each mix proportion. During the test, load was applied and released uniformly and continuously at a rate of 0.5 MPa/s, and micrometer readings (precision: 0.001 mm) were recorded.

#### 4.3.2. DIC Analysis Test

DIC is a full-field displacement measurement technique in which speckle pattern displacements are tracked. The experimental setup is composed of four essential components: a high-resolution digital camera mounted on a vibration-isolated tripod, uniform LED illumination sources, a computer workstation for system control, and specialized DIC 3D 2014a analysis software. Prior to testing, the specimen surface is prepared by applying a uniform layer of matte white paint as a base coat, followed by the spraying of black speckles to form a random speckle pattern. Critical system calibration is performed by aligning the camera optical axis perpendicular to the specimen surface, optimizing lighting conditions to achieve uniform illumination, and adjusting camera focus and aperture to ensure clear speckle patterns. During testing, sequential images are captured automatically at programmed intervals and are processed using DIC algorithms to compute full-field strain distributions by tracking sub-pixel displacements of the speckle pattern.

#### 4.3.3. AE Analysis Test

The AE measurement equipment is composed of a Physical Acoustics PCI-2 system with four 150 kHz resonant sensors. The sensors were coupled using Vaseline and secured with plastic tape. The AE system is activated when the threshold reaches 45 dB, and a gain of 40 dB is provided for the triggered AE signal. Time–frequency analysis of AE parameters, including amplitude, energy, and b-value, combined with mechanical data, was used to enable precise identification of crack initiation and classification of fracture modes.

## Figures and Tables

**Table 1:** Failure modes of different specimens.

Fine gangue content (%)	20	30	40	50	60
Failure mode	I	I	I	I	I
Mass concentration (%)	84	85	86	87	88
Failure mode	I	I	I	II	III
Binder content (%)	30	31	32	33	34
Failure mode	I	I	I	II	III

**Table 2:** Uniaxial compressive strength and elastic modulus of the backfill material.

Specimens	Compressive Strength (MPa)	Elastic Modulus (GPa)	Specimens	Compressive Strength (MPa)	Elastic Modulus (GPa)
GMJ-20	12.9 ± 0.42	6.4 ± 0.19	MGJ-87	16.8 ± 0.55	8.6 ± 0.27
GMJ-30	13.8 ± 0.45	6.8 ± 0.21	MGJ-88	17.5 ± 0.58	9.3 ± 0.29
GMJ-40	14.2 ± 0.47	7.1 ± 0.23	JGM-30	12.5 ± 0.40	6.1 ± 0.18
GMJ-50	14.0 ± 0.44	6.9 ± 0.22	JGM-31	13.3 ± 0.43	6.6 ± 0.20
GMJ-60	11.1 ± 0.36	5.6 ± 0.17	JGM-32	14.2 ± 0.46	7.1 ± 0.22
MGJ-84	9.1 ± 0.29	5.1 ± 0.15	JGM-33	15.7 ± 0.51	7.7 ± 0.24
MGJ-85	13.0 ± 0.42	6.5 ± 0.20	JGM-34	16.9 ± 0.56	9.0 ± 0.28
MGJ-86	14.2 ± 0.46	7.1 ± 0.22	Matrix	20 ± 0.64	10 ± 0.31

**Table 3:** Physical properties of FA and GGBS.

Properties	Density (g/cm^3^)	Specific Surface Area (m^3^/kg)	Moisture Content (%)	Loss on Ignition (%)	Average Particle Size (μm)
FA	2.14 ± 0.03	420 ± 8	0.61 ± 0.04	2.52 ± 0.12	29.71 ± 1.35
GGBS	2.88 ± 0.04	426 ± 7	0.10 ± 0.01	0.36 ± 0.03	9.60 ± 0.48

**Table 4:** Chemical compositions of FA and GGBS (wt%).

Chemical	CaO	SiO_2_	Al_2_O_3_	Fe_2_O_3_	Na_2_O	MgO	SO_3_	K_2_O
FA	3.35	52.78	34.18	5.64	0.15	0.31	0.49	1.65
GGBS	36.71	34.21	19.48	0.38	0.18	6.12	1.05	0.38

**Table 5:** Mix proportions of specimens.

Group	FA	GGBS	NaSiO_3_	NaOH	Water	Fine Aggregate	Coarse Aggregate	Fine Gangue Content	Mass Concentration	Binder Content
	kg/m^3^	kg/m^3^	kg/m^3^	kg/m^3^	kg/m^3^	kg/m^3^	kg/m^3^	%	%	%
ZGMJ-40 ZMGJ-86 ZJGM-32	638.4	33.6	231.6	37.3	133.3	410.3	615.4	40	86	32
ZGMJ-20	638.4	33.6	231.6	37.3	133.3	205.1	820.6	20	86	32
ZGMJ-30	638.4	33.6	231.6	37.3	133.3	307.7	718.0	30	86	32
ZGMJ-50	638.4	33.6	231.6	37.3	133.3	512.9	512.9	50	86	32
ZGMJ-60	638.4	33.6	231.6	37.3	133.3	615.5	410.3	40	86	32
ZMGJ-84	638.4	33.6	231.6	37.3	175.3	393.5	590.3	40	84	32
ZMGJ-85	638.4	33.6	231.6	37.3	154.3	401.9	602.9	40	85	32
ZMGJ-87	638.4	33.6	231.6	37.3	112.3	418.7	628.1	40	87	32
ZMGJ-88	638.4	33.6	231.6	37.3	91.3	427.1	640.7	40	88	32
ZJGM-30	598.5	31.5	217.1	35.0	143.4	429.8	644.7	40	86	30
ZJGM-31	618.5	32.6	224.4	36.1	138.4	420.1	630.0	40	86	31
ZJGM-33	658.4	34.7	238.9	38.4	128.3	400.6	600.8	40	86	33
ZJGM-34	678.3	35.7	246.1	39.6	123.3	390.8	586.2	40	86	34

**Table 6:** Mechanical properties of rock-like specimens and sandstone.

Mechanical Characteristics	Rock-Like Specimens	Sandstone [51]
Compressive strength (MPa)	17.3 ± 0.8	11–47.3
Elastic modulus (GPa)	5.6 ± 0.3	2.9–31.4

**Table 7:** Specimen size and quantity.

Test Name	Quantity	Specimen Size (mm)
**Uniaxial compression test**	3 × 13	100 × 100 × 300
**Elastic modulus test**	3 × 13	100 × 100 × 300
**Microscopic experiment**	3 × 10	100 × 100 × 100

## Data Availability

The raw data supporting the conclusions of this article will be made available by the authors on request.
